# Single-cell RNA and transcriptome sequencing profiles identify immune-associated key genes in the development of diabetic kidney disease

**DOI:** 10.3389/fimmu.2023.1030198

**Published:** 2023-03-29

**Authors:** Xueqin Zhang, Peng Chao, Lei Zhang, Lin Xu, Xinyue Cui, Shanshan Wang, Miiriban Wusiman, Hong Jiang, Chen Lu

**Affiliations:** ^1^Department of Nephropathy, People’s Hospital of Xinjiang Uygur Autonomous Region, Urumuqi, Xinjiang Uygur Autonomous Region, China; ^2^Department of Cardiology, People’s Hospital of Xinjiang Uygur Autonomous Region, Urumuqi, Xinjiang Uygur Autonomous Region, China; ^3^Department of Endocrine, People’s Hospital of Xinjiang Uygur Autonomous Region, Urumuqi, Xinjiang Uygur Autonomous Region, China; ^4^Department of Rheumatology Immunology, People’s Hospital of Xinjiang Uygur Autonomous Region, Urumuqi, Xinjiang Uygur Autonomous Region, China; ^5^Nephrology Clinical Research Center, People’s Hospital of Xinjiang Uygur Autonomous Region, Urumuqi, Xinjiang Uygur Autonomous Region, China; ^6^Department of Nephropathy, The First Affiliated Hospital of Xinjiang Medical University, Urumuqi, Xinjiang Uygur Autonomous Region, China

**Keywords:** diabetic kidney disease (DKD), single-cell RNA and transcriptome sequencing, immune cells, diagnostic markers, WGCNA (weighted gene co- expression network analyses)

## Abstract

**Background:**

There is a growing public concern about diabetic kidney disease (DKD), which poses a severe threat to human health and life. It is important to discover noninvasive and sensitive immune-associated biomarkers that can be used to predict DKD development. ScRNA-seq and transcriptome sequencing were performed here to identify cell types and key genes associated with DKD.

**Methods:**

Here, this study conducted the analysis through five microarray datasets of DKD (GSE131882, GSE1009, GSE30528, GSE96804, and GSE104948) from gene expression omnibus (GEO). We performed single-cell RNA sequencing analysis (GSE131882) by using CellMarker and CellPhoneDB on public datasets to identify the specific cell types and cell-cell interaction networks related to DKD. DEGs were identified from four datasets (GSE1009, GSE30528, GSE96804, and GSE104948). The regulatory relationship between DKD-related characters and genes was evaluated by using WGCNA analysis. Gene Ontology (GO) and Kyoto Encyclopedia of Genes and Genomes (KEGG) datasets were applied to define the enrichment of each term. Subsequently, immune cell infiltration between DKD and the control group was identified by using the “pheatmap” package, and the connection Matrix between the core genes and immune cell or function was illuminated through the “corrplot” package. Furthermore, RcisTarget and GSEA were conducted on public datasets for the analysis of the regulation relationship of key genes and it revealed the correlation between 3 key genes and top the 20 genetic factors involved in DKD. Finally, the expression of key genes between patients with 35 DKD and 35 healthy controls were examined by ELISA, and the relationship between the development of DKD rate and hub gene plasma levels was assessed in a cohort of 35 DKD patients. In addition, we carried out immunohistochemistry and western blot to verify the expression of three key genes in the kidney tissue samples we obtained.

**Results:**

There were 8 cell types between DKD and the control group, and the number of connections between macrophages and other cells was higher than that of the other seven cell groups. We identified 356 different expression genes (DEGs) from the RNA-seq, which are enriched in urogenital system development, kidney development, platelet alpha granule, and glycosaminoglycan binding pathways. And WGCNA was conducted to construct 13 gene modules. The highest correlations module is related to the regulation of cell adhesion, positive regulation of locomotion, PI3K-Akt, gamma response, epithelial-mesenchymal transition, and E2F target signaling pathway. Then we overlapped the DEGs, WGCNA, and scRNA-seq, SLIT3, PDE1A and CFH were screened as the closely related genes to DKD. In addition, the findings of immunological infiltration revealed a remarkable positive link between T cells gamma delta, Macrophages M2, resting mast cells, and the three critical genes SLIT3, PDE1A, and CFH. Neutrophils were considerably negatively connected with the three key genes. Comparatively to healthy controls, DKD patients showed high levels of SLIT3, PDE1A, and CFH. Despite this, higher SLIT3, PDE1A, and CFH were associated with an end point rate based on a median follow-up of 2.6 years. And with the gradual deterioration of DKD, the expression of SLIT3, PDE1A, and CFH gradually increased.

**Conclusions:**

The 3 immune-associated genes could be used as diagnostic markers and therapeutic targets of DKD. Additionally, we found new pathogenic mechanisms associated with immune cells in DKD, which might lead to therapeutic targets against these cells.

## 1 Introduction

Type I or II diabetes can cause diabetic kidney disease (DKD), which is regarded as a microvascular complication that poses a grave threat to human health. Over fifty percent of individuals with end-stage renal disease (ESRD) caused by DKD who are getting renal replacement therapy (RRT) in the majority of nations are affected by diabetes (1). Because of improved diabetes management, there has been a decline in the incidence of DKD over the past 30 years. However, the risk of renal failure remains high(2–5). According to a previous study, about 30% of people with type 1 diabetes and 40% of people with type 2 diabetes turn up microvascular complications (1, 6). DKD occurs in families across various populations, suggesting a genetic predisposition (7, 8). For this reason, it is essential to get a more in-depth knowledge of the pathophysiology of DKD so that novel therapeutic techniques may be developed to stop, halt, and even reverse the progression of DKD. DKD, as a multifactorial illness, includes a complicated interaction of hemodynamic and metabolic variables, such as hyperglycemia, renin-angiotensin-aldosterone system activation, and advanced glycation end-products (9). DKD can be predicted with low sensitivity and specificity by microalbuminuria, which is a biomarker of early diabetes (10). The presence of micro/macro-albuminuria is not always related to DKD. Many diabetic patients have decreased renal function when considerable proteinuria is absent (11). Moreover, DKD is still diagnosed with renal biopsy (12). However, it is an invasive procedure associated with complications such as infection and hemorrhage (13). Additionally, it is impossible to proceed continuously as the kidney disease progresses, and sampling errors are highly likely. Hence, in order to predict DKD development, it is essential to investigate noninvasive and sensitive immune-associated biomarkers.

As a result of the “bulk” RNA-seq analyses performed over the last 20 years, we now have a better understanding of the transcriptional landscape of kidneys, which only describes the average transcriptome in bulk renal tissue or even in high-resolution compartmentalized kidneys though highly informative, therefore masking or skewing the signals of the transcriptome (14–16). It is possible to determine particular disease-causal cells and genes by Single-cell RNA sequencing(scRNA-seq), which cooperates with the definition of cell types and the status of gene expression in given cells. Recent years have significantly improved scRNA-seq’s sensitivity, accuracy, and efficiency (17). ScRNA-seq offers advantages over RNA-seq, including dissecting heterogeneity within cell populations and identifying rare cells related to the disease by using the single-cell profiles in mixed-cell populations(18). It is found that there are dynamic changes in gene expression of experimental diabetic kidney diseases by scRNA-seq of glomerular cells (19). Xi Lu et al. identified the role of immune cells and their marker genes as related key pathophysiologic items in DKD development by scRNA-seq (20). When compared to DEGs, WGCNA is a systems biology to investigate correlations between genes across microarray samples. A weighted gene co-expression network could be displayed with WGCNA, identifying module-related genes and exploring the relationship between genes and phenotypes. Disease-related genes could be identified through rational analysis of these modules (21). It was uncovered that there are six DKD-related candidate targets by WGCNA and DEG analysis of DKD datasets in the study of Chen J et al. (22). Herein, we combined three methods containing scRNA-seq, DEG, and WGCNA to identify candidate DKD-related gene targets regulators. By studying the mechanism of DKD development and identifying potential therapeutic targets, we aimed to advance our understanding of the disease.

## 2 Materials and methods

### 2.1 Data acquisition

**Figure 1:**
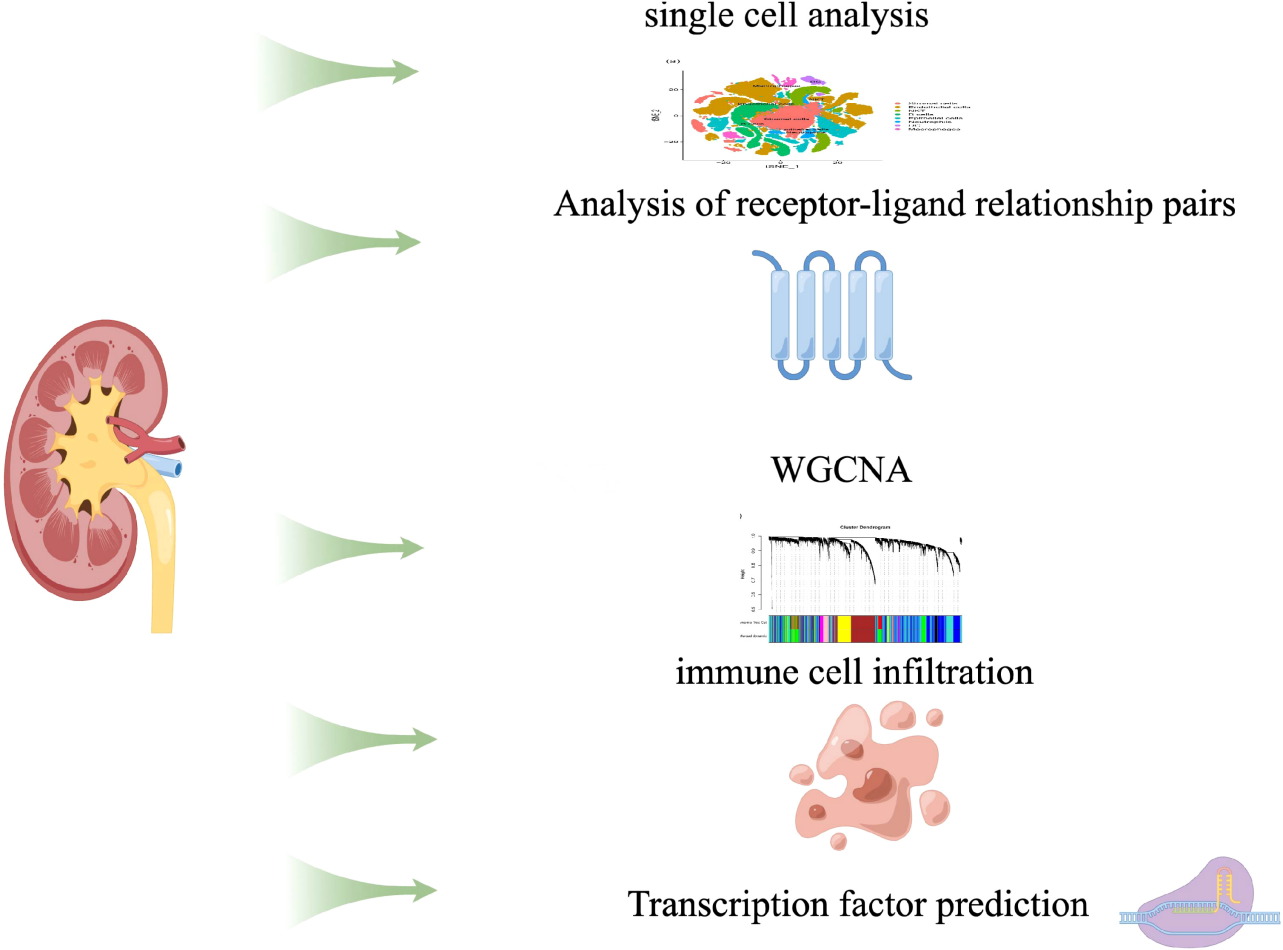
Workflow of this study.

National Center for Biotechnology Information (NCBI) creates and maintains GENE EXPRESSION OMNIBUS (GEO) database (https://www.ncbi.nlm.nih.gov/geo/info/datasets.html), which is a gene expression database. Data from international research institutions has been submitted to the database since 2000. Data from microarrays, next-generation sequencing, and other high-throughput sequencing experiments are stored in the GEO database. We gained GSE131882 from the GEO public database Series Matrix data file in NCBI, of which annotation platform is GPL24676. scRNA analysis was conducted through 6 copies (Control=3, DKD=3) of DKD-related data with complete expression profiles downloaded. Meanwhile, we download the Series Matrix File data file of GSE1009 from the GEO public database, the ontology platform is GPL8300, and 6 copies (Control=3, DKD=3) of DKD-related data with complete expression profiles were downloaded for this analysis. The Series Matrix File data file of GSE30528 was obtained from the GEO public database in NCBI, and the annotation platform is GPL571, and 22 (Control=13, DKD=9) DKD-related data with complete expression profiles were download for this analysis. The Series Matrix File data file of GSE96804 was gained from the GEO public database in NCBI, the annotation platform is GPL17586, and 61 (Control=20, DKD=41) DKD-related data with complete expression profiles were download for this analysis. The Series Matrix File data file of GSE104948 was obtained with the same method, the annotation platform is -GPL22945, and 25 (Control=18, DKD=7) DKD-related data with complete expression profiles were downloaded for this analysis. The Series Matrix File data file of GSE104948 could be gotten from the same public database web, the annotation platform is GPL24120, and 8 (Control=3, DKD=5) DKD-related data with complete expression profiles were downloaded for this analysis. The workflow of this study is shown in **Figure 1**.

### 2.2 Single cell analysis

In this analysis, the exon, inex and intron results of each sample were taken as a single sample to get an expression matrix of 373942 cells * 15398 features. We set the parameters min.cells=3 and min.features=50 to read in the expression spectrum, and got a single cell object of 111,360 cells. The low-expression cells of this object were screened by nFeature_RNA > 50 & percent.mt < 5, and finally a single cell object with 15398 features of 110544 cells was obtained. The data were standardized by NormalizeData function, and the 10 genes with the highest standardized variance were marked. ScaleData and RunPCA are used in turn to standardize the data and PCA analysis, in which the parameter npcs = 20 is set in PCA analysis. Then, the best PC value of this analysis is selected by ElbowPlot and JackStraw results. Selecting the best PC value of 15 and getting the Cluster and tSNE values through FindNeighbors, FindClusters and RunTSNE in turn. Setting the parameter min.PCT = 0.25 to get the unique markers gene of each Cluster through FindAllMarkers, and selected the top 10 gene expression levels of avg_log2FC in each Cluster for heat map display. At last, we annotated the Cluster cell subtypes by SingleR with ImmGenData as the annotation ref file and label.main as labels, and counted the number of cell sample contained in each subtype. FindAllMarkers method was used to obtain the differential genes of each cell subtype for further analysis.

### 2.3 Ligand-receptor interaction analysis

CellPhoneDB is an open acquired database of curated receptors, ligands and their interactions. Both ligands and receptors contain subunit structures that accurately represent heteromeric complexes. The ligand-receptor database of CellPhoneDB is integrated with UniProt, Ensembl, PDB, IUPHAR, etc., and stores a total of 978 proteins, which can comprehensively and systematically analyze the communication molecules between cells and study the mutual communication and communication between different cell types. By calling the statistical analysis of the software package CellphoneDB, a significant analysis of the ligand-receptor relationship of the features in the single-cell expression profile was performed. The cluster labels of all cells were set to be randomized 1000 times to determine the mean of the mean expression levels of receptors and ligands in the clusters or interacting clusters. This yields a null distribution (also known as Bernoulli, two-point distribution) for each receptor-ligand pair in each pair comparison between the two cell types. Finally, some interesting ligand-receptor pairs were selected for the display of relational pairs.

### 2.4 WGCNA analysis

**Figure 2:**
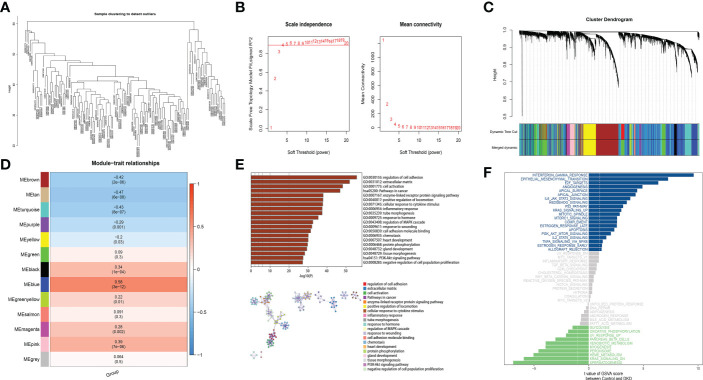
A coexpression module has been constructed by analyzing WGCNA and Metascape functional enrichment scores for MEblue genes. **(A)** Clustering of module hub genes in a hierarchical manner that summarizes the modules that were identified in the clustering analysis. **(B)** The scale independence plot, mean connectivity plot, and scale-free topology plots, 4 was an appropriate soft-power. **(C)** The cluster dendrogram shows the modules that make up the co-expression network. **(D)** Analysis of connection of the modules with immune scores. **(E)** GO and KEGG analysis of the model genes. **(F)** Differential pathway enrichment between DKD and control.

We conduct a weighted network to identify co-expressed gene modules, the core genes of a network, and the connection between genes and phenotypes. Furthermore, WGCNA-R package was applied to build the co-expression networks of genes in the gene set, In this analysis, soft threshold power value was determined for constructing a scale-free topology network. More specifically, We used the “sft$powerEstimate” function to perform the analysis of the network topology and got the relevant values corresponding to the alternative soft thresholds(shown on **Figure 2B**). We set the height to 0.9, and the minimum candidate threshold to reach this height is 4. Therefore, we chose a soft threshold of 4.

Based on the weighted adjacency matrix, the topological overlap matrix (TOM) was constructed to perform communication network, and creating the clustering tree structure of the TOM matrix was done by applying hierarchical clustering. Each branch of the cluster tree represents a divers gene module, and each color displays a different gene module. Each gene is classified into modules on the basis of the weighted correlation coefficient, and genes with similar expression patterns are grouped together. The gene expression patterns of thousands of genes divide them into variety modules.

### 2.5 Gene function enrichment analysis

Analyzing and visualizing the Metascape database (www.metascape.org) allowed us to get the biological functions and signal pathways related to disease occurrence and development. We combined Gene Ontology (GO) analysis with Kyoto Encyclopedia of Genomes (KEGG) analysis to explore specific genes. Min overlap≥3 & p ≤ 0.01 is regarded as statistical significance. In addition, clusterProfiler was used to annotate the function of the key genes in order to identify their functional relevance. It was determined that pathways enriched with both p- and q-values below 0.05 within GO and KEGG were significant.

### 2.6 Immune cell infiltration analysis

RNA-Seq data from DKD patients and healthy controls were analyzed using the CIBERSORT algorithm in order to determine the percentage of immune infiltrating cells in each group, and perform spearman connection analysis about immune cells and gene expression.

### 2.7 GSEA analysis

A GSEA analysis compares gene expression levels in two types of samples based on a predetermined set of genes, and ranks these genes based on the level of different expression, the predefined gene set is tested to see if it is enriched near the top or bottom of the sequence in the further study. GSEA was utilized in this work to compare the variations in the KEGG signaling pathway between the high and low expression groups, and investigate the molecular mechanisms of the key genes in the two groups based on 1000 substitutions and phenotypic substitutions.

### 2.8 Gene Set Variation Analysis (GSVA)

Transcriptome sequencing enrichment can be evaluated using gene set variation analysis (GSVA), which is a non-parametric and unsupervised approach. As GSVA scores the genes of interest comprehensively, pathway-level changes are converted into gene-level changes, and study the biological function. The sample of gene sets was obtained from the molecular signature database (v7.0), and a comprehensive score would be assigned to each gene set to determine whether biological function has changed between samples using the GSVA algorithm.

### 2.9 Enrichment of transcription factors motifs related to the key genes

This study used the R package “RcisTarget” to predict transcription factors. All calculations performed by RcisTarget were based on motifs. The normalized enrichment score (NES) for motifs depended on the motifs amount in the database. In addition, the motifs annotated by annotation files, which were inferred further based on motif similarity and gene sequence. The first step in estimating the overexpression of each motif on a gene set was to compute the area under the curve (AUC) of the motif-motif set pair. This was calculated from the recovery curves of the gene sets for motif ordering. The NES for each motif was estimated according to the AUC distribution of all motifs in the gene set. The rcistarget.hg19.motifdb.cisbpont.500bp was used for the Gene-motif rankings database.

### 2.10 The clinical prospective cohort design and sample collection

We recruited 35 patients with DKD and 35 healthy controls between 2019 and 2021 at the People’s Hospital of the Xinjiang Uygur Autonomous Region. In a cohort of 35 patients with DKD, All patients met the following diagnostic criteria:(1)Patients aged ≥30 years who underwent clinical kidney biopsy;(2)patients had a pathological diagnosis with DKD as the only kidney disease diagnosis;(3)patients who had an eGFR ≥60 mL/min/1.73 m2. The outcome of interest was development of DKD defined as a composite of new onset ESKD (dialysis, kidney transplantation or death from renal cause), a doubling of serum creatinine or a decrease of eGFR by ≥50%. The occurrence of DKD development was ascertained by the medical records (23). Based on 2.6-year median follow-up data, key genes were examined in relation to DKD development. Patient informed consent was obtained, and the study was approved by an institutional ethical committee at the People’s Hospital of Xinjiang Uygur Autonomous Region(Number KY 2019062614). Blood samples were collected after subjects fasted for at least 12 hours before blood was drawn. Information from relevant medical records was recorded and coded. The participants provided a blood sample of 3 mL, which was collected in Eppendorf tubes without anticoagulants and allowed to stand for one hour at 4°C. The supernatant containing the serum proteins was aliquoted into 0.5 mL aliquots and kept at 80°C in a refrigerator for future use after centrifugation at 3000 rpm at 4°C for 15 minutes (24). At the same time, we obtained the renal tissue samples of these participants, which were obtained by percutaneous renal puncture. The remaining samples after clinical diagnosis were kept for our research.

### 2.11 ELISA Validation

The collection of kidney tissue samples followed conventional hospital protocol.

Samples of kidney tissue were kept at -80°C. The levels of SLIT3, PDE1A, and CFH were measured using an enzyme linked immunosorbent assay method in accordance with the manufacturer’s instructions (Keweinuo Systems, Inc., Xiuyuan, Jiangsu, China, the batch numbers are: KWN-162079, ZY-PDE1A-Hu and JL11886).For the quantitative measurement of SLIT3,PDE1A, and CFH concentrations, the manufacturer-recommended quantitative sandwich enzyme immunoassay method was used. On a microplate, a monoclonal antibody specific for SLIT3, PDE1A, and CFH were coated beforehand. Pipetted into the wells were standards and samples, and any SLIT3, PDE1A, and CFH present was bound by the immobilized antibody. After removing any unattached compounds, an anti-SLIT3, anti-PDE1A and anti-CFH enzyme-linked polyclonal antibody were applied to the wells. After removing any unbound antibody-enzyme reagent with a wash, a substrate solution was added to the wells, and color developed in proportion to the quantity of SLIT3, PDE1A, and CFH bounds in the first stage. The color development was halted, and the color’s intensity was assessed. The standards of recombinant human SLIT3, PDE1A, and CFH were measured to create a standard curve. Results were reported in mg/L units (25).

### 2.12 Immunohistochemistry (IHC)

After signing the consent of participants, the right lower renal pole renal biopsy was performed under the guidance of ultrasound. Take the prone position, after anaesthetizeing participants. Taking the No.20 puncture trocar, and pierced the skin along the local anesthesia point, and gradually penetrated into the renal capsule under the detection of ultrasound, carefully broke through the renal capsule, and instructed the patient to hold his breath, pulled the trigger, and pulled out the needle. Using the remaining samples after diagnosis, fixed them overnight, dehydrated them with 30% sucrose for one day (or the samples sank to the bottom), and sliced them immediately. Then we cleaned the cut slides with TBS, and stored in TBS for 4 degrees for a short time and incubated with 1 ml of 0.5% Triton X-100 for 1 hour subsequently. Our samples were sealed with serum at RT, pH7.4 for 2h. Tissue sections were incubated overnight at 4 °C with rabbit monoclonal antibodies against SLIT3 (1:200), PDE1A (1:500) and CFH (1:2000) (Keweinuo Systems, Inc., Xiuyuan, Jiangsu, China; the batch numbers are: abx317205, abs138818 and abx124187). After rewarming at room temperature for 30 minutes, the slices were washed with PBS for at least 3 times. Then it was incubated with goat anti-rabbit immunoglobulin G (IgG) (1:2000) for 20 minutes at 37 °C, followed by three times PBS washes. The chips were dyed with hematoxylin (PT001, Zhenjiang Xiuyuan Biotechnology Co., Ltd., Zhenjiang, China) for 1 minute, then turned blue in 1% ammonia water and washed with water. After dehydration with ethanol series, the slices were washed in xylene and sealed with neutral glue. We performed quantitative analysis of IHC results using imageJ software, a free software developed by the NIH that is widely used in biomedical applications.

### 2.13 Western blot

Kidney tissues were added into 1 mL cell lysis solution for cell lysis. protein sample was mixed with 10% SDS gel loading buffer at 4 °C for 4 minutes, and boiled at 100 °C for 10 minutes. After that, protein was separated by electrophoresis and transferred to nitrocellulose membrane. After being blocked overnight with 5% skim milk at 4 °C, the membrane was mixed with rabbit anti-SLIT3(1∶2000), anti-PDE1A(1∶1000), anti-CFH(1∶2000) and GAPDH(1∶10) (Keweinuo Systems, Inc., Xiuyuan, Jiangsu, China; the batch numbers are: abx317205, abs138818,abx124187 and H00002597-PW2), and then with goat anti-rabbit IgG labeled with horseradish peroxidase (1:10,000) at 37 °C. After washing with PBS buffer for 3 times at room temperature for 10 minutes each time, the film was immersed in the enhanced chemiluminescence reaction solution for 5 minutes at room temperature and exposed to GAPDH as the internal reference. The ratio of the gray value of the target band to the gray value of the internal reference band was regarded as the relative expression level of protein.

### 2.14 Statistical analysis

In this analysis, R language (version 4.0) was used. A compared t-test was used to determine whether there was a statistical difference between the normalized expressions of the genes screened. ANOVA was used to explain the differences between different groups of IHC score. Using the Kaplan-Meier method, DKD development curves for different genes expression levels were generated using graphpad (version 9.0), and log-rank tests were used to compare them. During the median follow-up time of 2.6 years from diagnosis to endpoint, we investigated the influence of hub gene expression levels on kidney function development among DKD patients. It was regarded statistically remarkable at p<0.05 in all tests that were two-sided. Spearman method was used to study the linear relationship between protein expression for ELISA and IHC score.

## 3 Results

### 3.1 Preprocessing of single-cell expression profiling data

In this analysis, the expression profile was used to include 6 samples, including 3 healthy kidney tissue samples and 3 kidney tissue samples from patients with DKD, and the expression level of a total of 111,360 cells was detected (**Figures S1A**, **S1B**). Feature expression profiles of 110,544 cells with number of the features of RNA more than 50 and percent.mt below 5 in the expression profile were selected and included for subsequent analysis. The level of gene set was displayed and the top 10 genes were marked (**Figure S1C**).

### 3.2 Single-cell sample subtype clustering analysis

**Figure 3:**
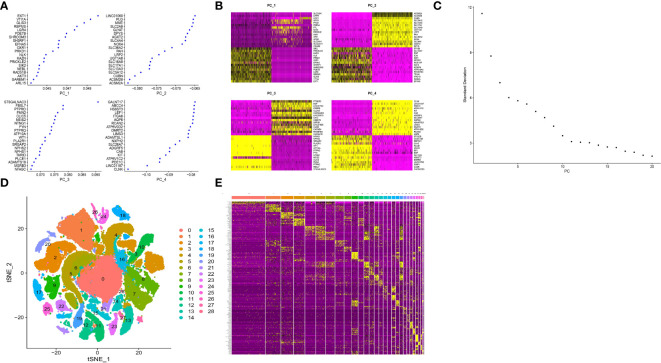
Cluster analysis of single cell sample subtypes. **(A)** Reduced maintenance number of main genes of PC1, PC2, PC3 and PC4. **(B)** The scores of cell genes on PC1, PC2, PC3 and PC4. **(C)** Elbowplot for identifying the optimal PCs. **(D)** TSNE dimensionality reduction of 20 genes in the samples. **(E)** Heat map of gene expression.

PCA dimensionality reduction analysis was performed and it was found that they have different score values ​​in different dimensions (**Figures 3A, B**). However, PCA dimensionality reduction analysis between samples revealed that the overall differences between samples were not significant (**Figure S2**). The optimal number of pcs was observed by ElbowPlot: 15 (**Figure 3C**), and finally 29 subtypes were obtained by TSNE (**Figure 3D**). We found a large number of genes with significant differences in expression levels between these subtypes. Last but not least, we showed the expression levels of 10 genes that differed the most between subtypes (**Figure 3E**).

### 3.3 Annotation of cluster subtypes

**Figure 4:**
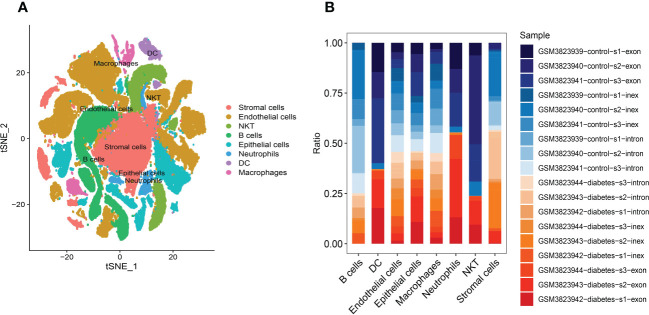
Annotation of cluster subtypes **(A)** 29 clusters were annotated to 8 cell categories: Stromal cells, Endothelial cells, NKT, B cells, Epithelial cells, Neutrophils, DC and Macrophages middle. **(B)** Samples in each cell subtype.

ImmGenData was used as the annotation data to annotate each subtype through the R package SingleR, and 29 clusters were annotated to 8 cell categories: Stromal cells, Endothelial cells, NKT, B cells, Epithelial cells, Neutrophils, DC and Macrophages middle (**Figure 4A**). Although the amount of detected cells in the DKD and control groups was not 1:1 in each cell subtype, both DKD and control samples were included in different cell types (**Figure 4B**). For example, B cells and NKT subtypes contain more cells from healthy tissues. Finally, a total of 1,193 cell subtype Marker genes were extracted (**Supplement table 1**) from single-cell expression profiles by FindAllMarkers.

### 3.4 Analysis of receptor-ligand relationship pairs

**Figure 5:**
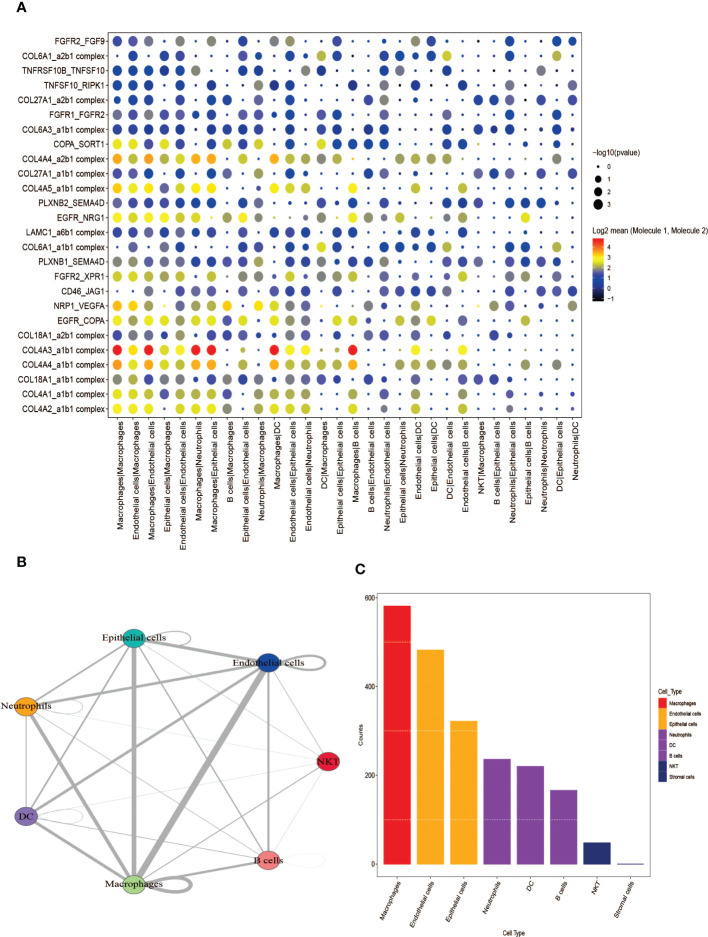
Analysis of receptor-ligand relationship pairs. **(A)** Receptor-ligand trafficking and intracellular signaling. **(B)** Interaction numbers between cell groups. **(C)** The number of ligand-receptor gene pairs corresponding to each cell group.

The software package CellphoneDB was applied for analyzing the ligand-receptor relationship of the features in the single-cell expression profile. Finally, some ligand-receptor pairs were selected for display (**Figure 5A**). It was found that Macrophages|Neutrophils, Macrophages|Epithelial cells had high interaction scores for their interactions with COL4A3_a1b1 complex, COL4A4_a2b1 complex. It was also found that the number of potential ligand-receptor pairs between cells such as Macrophages and other cells is extremely high (**Figure 5B**). Finally, the number of ligand-receptor gene pairs corresponding to p value < 0.05 in each cell group was counted and it was found that macrophages and Endothelial cells have more potential interactions with other cell subtypes, especially macrophages (**Figure 5C**).

### 3.5 Analysis of differential expression by RNA-seq

**Figure 6:**
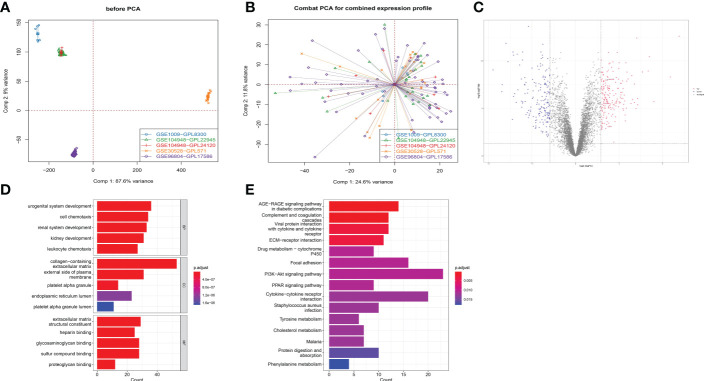
Functional analysis of different expression genes in RNA-sequencing. **(A)** Five GEO data sets were combined into expression profiles of 122 samples by ComBat. **(B)** Combat PCA for combined expression profile. **(C)** Volcano plot displaying differential expressed genes (DEGs) between DKD patients and healthy control for combined expression profile. **(D)** Gene Ontology plots of over-expressed and under-expressed terms. **(E)** Kyoto Encyclopedia of Genes and Genomes (KEGG) analysis.

The five GEO datasets, namely GSE1009, GSE30528, GSE96804, GSE104948 (GPL22945), and GSE104948 (GPL24120) were combined to the expression profiles of 122 samples through ComBat (control group: 57 cases; DKD group: 65 cases) (**Figures 6A, B**). After that, the limma package was used to count the differential genes between patients and normal samples. The differential gene screening conditions were: adj.P.Val<0.05 and |logFC| > 0.585 (26), and finally 178 up-regulated genes and 178 down-regulated genes were screened out (**Figure 6C**). The functional analysis on these 356 differential genes was performed and it was found that they were enriched in urogenital system development, kidney development, platelet alpha granule and glycosaminoglycan binding pathways (**Figures 6D, E**).

### 3.6 Co-expression modules of DKD and functional enrichment of modular genes

To perform the co-expression network of marker genes in the DKD sample, WGCNA analysis was carried out. The disease state was taken as a clinical trait and further used to construct a WGCNA network to screened biomarkers in the process of DKD. The soft threshold β, which was calculated by the function “sft$powerEstimate”, was set to 4 (**Figures 2A, B**). Hierarchical clustering trees (average-linked hierarchical clustering) were constructed from weighted correlation coefficients (TOM matrix) between genes, with different branches of the clustering trees being genes with similar patterns, representing different gene modules. Gene clusters that were not assigned to a specific module were defined as gray modules, 13 gene modules were identified in the analysis, including turquoise (n=2104), blue (n=1808), brown (n=1124), green (n=777), turquoise yellow (n=776), black (n=204), pink (n=177), magenta (n=138), purple (n=111), green-yellow (n=91), salmon (n=60), tan(n=65) and grey (n=8) modules (**Figure 2C**). The modules and traits were further analyzed and it was found that ME-blue modules had the most significant correlation with the sample traits (**Figure 2D**). Metascape functional enrichment analysis on genes in MEblue module was performed. Our results proved that GO and KEGG analysis mainly enriched signaling mechanisms concluding positive regulation of locomotion, regulation of PI3K-Akt signaling pathway and cell adhesion. The above pathways are closely related (**Figure 2E**). Through GSVA pathway analysis, it was found that many pathways enriched by these genes were significant between groups, such as INTERFERON GAMMA RESPONSE, EPITHELIAL MESENCHYMAL TRANSITION, E2F TARGETS and other pathways (**Figure 2F**).

### 3.7 Screening and verification of key genes.

**Figure 7:**
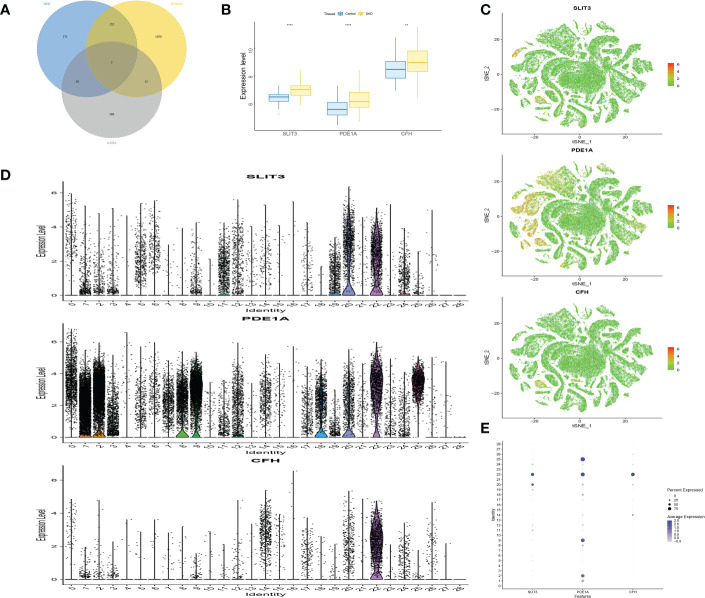
Screening and verification of key genes. **(A)** The Venn diagram displays the overlap genes obtained by three methods. **(B)** Box plots of the level of SLIT3, PDE1A and CFH in DKD and control samples. **(C)** The expression distribution of marker genes. **(D)** Violin plots showing the level of SLIT3, PDE1A and CFH in different identity. **(E)** Dot plot displaying the normalized mean level of markers. **P<0.01; ****P<0.0001.

It was found that only 3 genes in the ME-blue module had significant differences between groups in RNA-seq level and scRNA level expression, namely SLIT3, PDE1A and CFH (**Figure 7A**), where the between-group significance of **Figures 7B–E** was analyzed by wilcoxon test.

### 3.8 Assessment of immune infiltration levels

**Figure 8:**
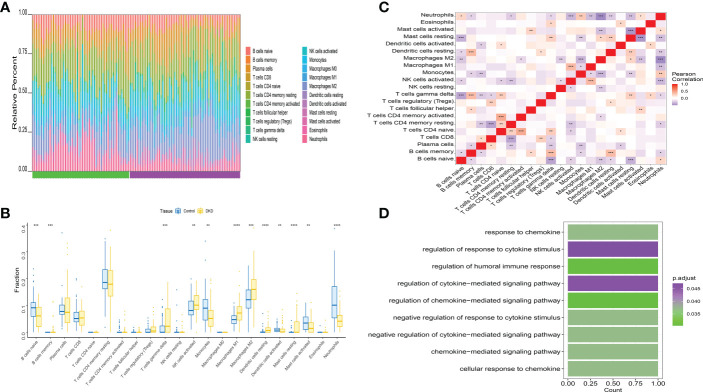
Distribution and visualization of immune cell infiltration. **(A)** The relative percentage of 22 types of immune cells. **(B)** Box plots demonstrating 22 immune cell subtypes between DKD and healthy controls. Blue represents normal and yellow represents DKD samples. **(C)** The heat map demonstrated the interaction of 21 kinds of immune cells. Red showed the positive relation and blue displayed the negative relation, The correlation parameter was shown with the number. **(D)** GO analysis of three genes. *P<0.05; **P<0.01; ***P<0.0001.

Immune microenvironment consist of inflammatory factors, immune cells, special physical and chemical characteristics, extracellular matrix, various growth factors, immune-related fibroblasts, and the diseased cells themselves. Diagnoses and survival of major diseases are greatly affected by the immune microenvironment. Analyzing key gene functions in major diseases and immune infiltration, a further investigation was conducted to determine the mechanisms and key genes associated with DKD. First, a significant study on the immune microenvironment score between the disease and the normal group found that immune microenvironment factors consist of B cells naive, B cells memory, T cells gamma delta, Macrophages M1, Macrophages M2, and Dendritic cells resting were remarkably higher in the group (**Figures 8A, B**). At the same time, it was found that there were multiple significant correlation pairs between the immune factors through correlation analysis (**Figure 8C**). We also supplemented the GO analysis of three genes as shown in **Figure 8D**, and the analysis results showed that SLIT3 and CFH were also involved in the immune response.

### 3.9 Analysis of the correlation between key genes and immune infiltration

**Figure 9:**
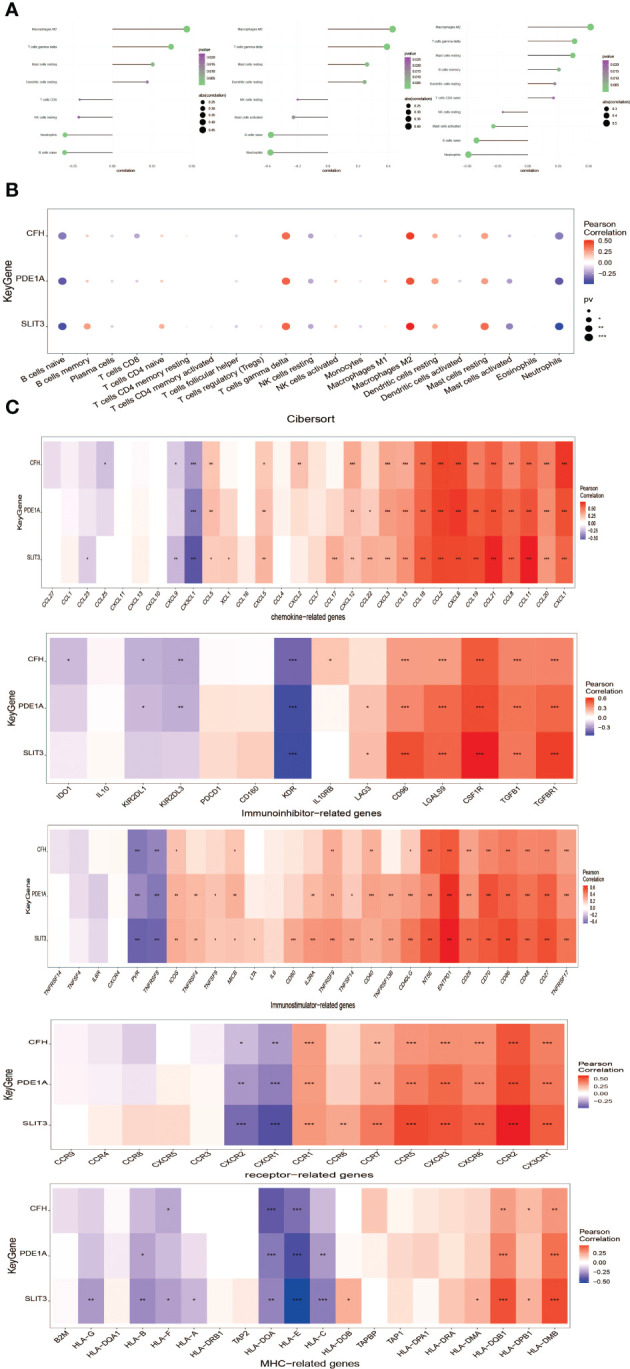
Correlation analysis of key genes and immune infiltration.**(A)** Interaction between core genes and immune cells. **(B)** Interaction between the level of core genes and immune cells abundance. **(C)** Connection between core genes and immunomodulators, chemokines and cell receptors. *P<0.05; **P<0.01; ***P<0.0001.

The Interaction between key genes and immune cells was further explored, and it was found that there were positive interactions between 3 core genes (SLIT3, PDE1A, and CFH) and immune cell infiltration, such as T cells gamma delta, Mast cells resting, Macrophages M2. However, B cells naive and Neutrophils were apparently negatively correlated with SLIT3, PDE1A, and CFH (**Figure 9A**). The correlations of the 3 key genes with various immune factors from the TISIDB database were further obtained, consist of chemokines, cellular receptors, and immune modulators (**Figure 9B**). Next the specific signaling pathways enriched by the three key genes and the potential molecular mechanisms of the core genes related to the development of DKD were investigated. Subsequently, we implemented the connection between 3 key genes and chemokine-related genes, immunoinhibitory-related genes, immunostimilator-genes, receptor-related genes, MHC-related genes using cibersort method. The results showed that the 3 key genes we obtained had closely relationship with chemokine-related genes, immunoinhibitory-related genes, immunostimilator-genes, receptor-related genes, MHC-related genes (**Figure 9C**).

### 3.10 GSEA analysis of key genes

**Figure 10:**
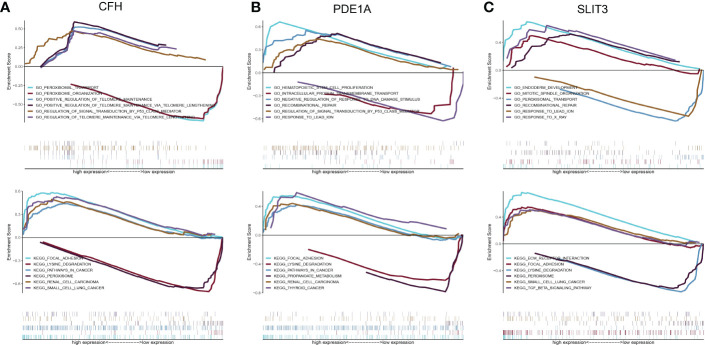
Discussion on the specific signaling mechanism of key genes. **(A)** GO and KEGG analysis of CFH gene using GSEA method. **(B)** GO and KEGG analysis of PDE1A gene using GSEA method. **(C)** GO and KEGG analysis of SLIT3 gene using GSEA method.

A further study was conducted to explore the specific signaling pathways that are enriched by the three key genes, as well as the molecular mechanisms underlying their effects on DKD development. Pathways with high significance were selected and displayed separately. The CFH gene GO enriched pathways included PEROXISOMAL TRANSPORT, PEROXISOME ORGANIZATION and other pathways, and KEGG enriched pathways included FOCAL ADHESION, LYSINE DEGRADATION and other pathways (**Figure 10A**). The PDE1A gene enriched pathways included HEMATOPOIETIC STEM CELL PROLIFERATION, INTRACELLULAR PROTEIN TRANSMEMBRANE TRANSPORT and other pathways. KEGG enriched pathways included FOCAL ADHESION, LYSINE DEGRADATION and other pathways (**Figure 10B**). SLIT3 gene GO enriched pathways included ENDODERM DEVELOPMENT, MITOTIC SPINDLE ORGANIZATION and other pathways. The pathways enriched by KEGG included ECM RECEPTOR INTERACTION, FOCAL ADHESION and other pathways (**Figure 10C**).

### 3.11 Regulatory network analysis of key genes

**Figure 11:**
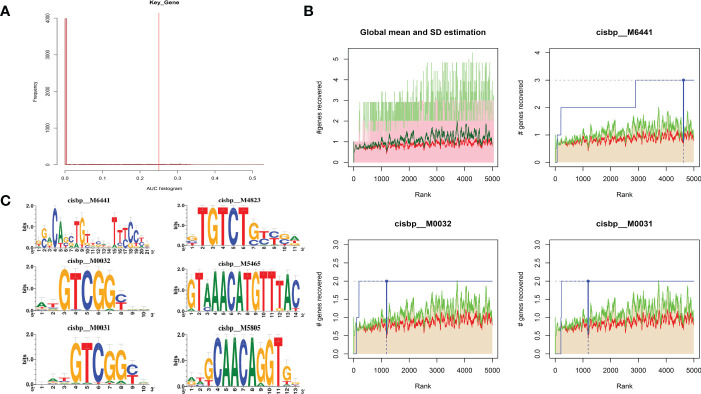
Analysis of regulatory network of key genes. **(A)** Motif-TF annotation based on normalized enrichment score. **(B)** Optimal gene based on motif enrichment. **(C)** Motif enrichment and its annotation information.

Using these three key genes for the gene set, and analyzing their regulation using multiple transcription factors, it was discovered that they are regulated by a common mechanism. Therefore, Cumulative recovery curves were used to enrich these transcription factors (**Figures 11A, B**). The analysis results illustrated that the Motif with the highest normalized enrichment score (NES: 5.75) was annotated as cisbp:M6441. It was found that three genes, CFH, PDE1A, and SLIT3, were enriched in the motif. Transcriptional factors of the core genes were identified in all enriched motifs (**Figure 11C**).

### 3.12 The study of disease gene expression levels

**Figure 12:**
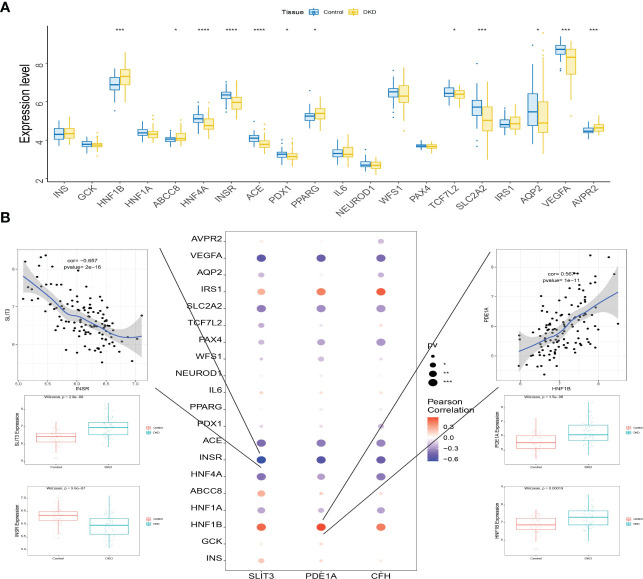
Connection between the level of key genes and several DKD-related genes. **(A)** Box plots displaying the expression of the expression of the top 20 genes related to DKD. **(B)** Interaction between the level of key genes and the expression of several DKD-related genes. *P<0.05; ***P<0.0001; ****P<0.0001.

A total of 3,421 DKD-related disease genes were obtained from the GeneCards database (https://www.genecards.org/). The levels of genes (**Figure 12A**) were analyzed, and it was discovered that the expression levels of key genes were remarkably related to the expression levels of multiple disease-related genes (**Figure 12B**).

### 3.13 Verification of key genes in clinical population

**Figure 13:**
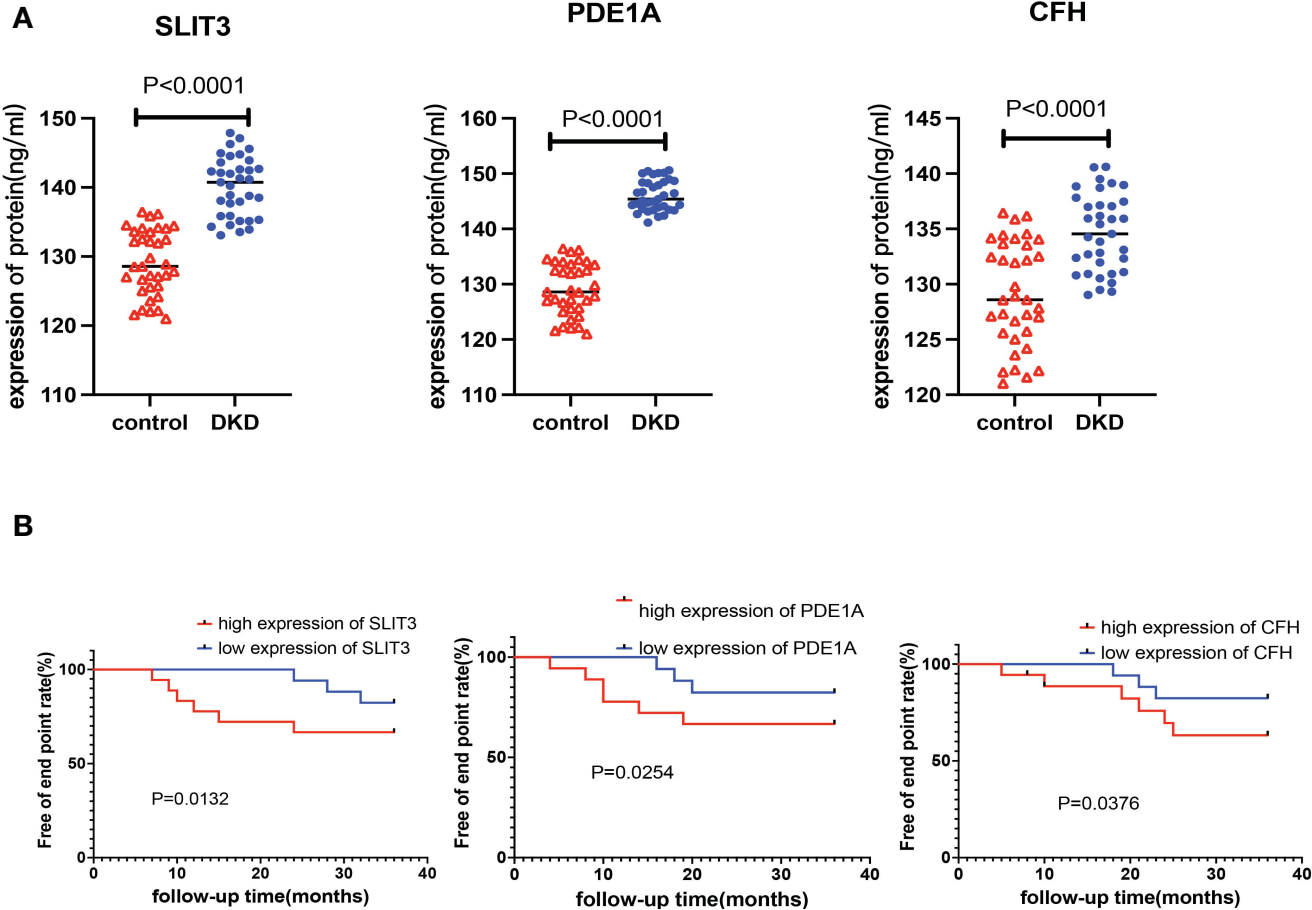
Verification of key genes in clinical population. **(A)** Expression levels of key genes in plasma samples of DKD patients and healthy control. **(B)** Kaplan-Meier chart to display the association between the key genes and DKD development rate of different expression level of the 3 genes.

**Figure 14:**
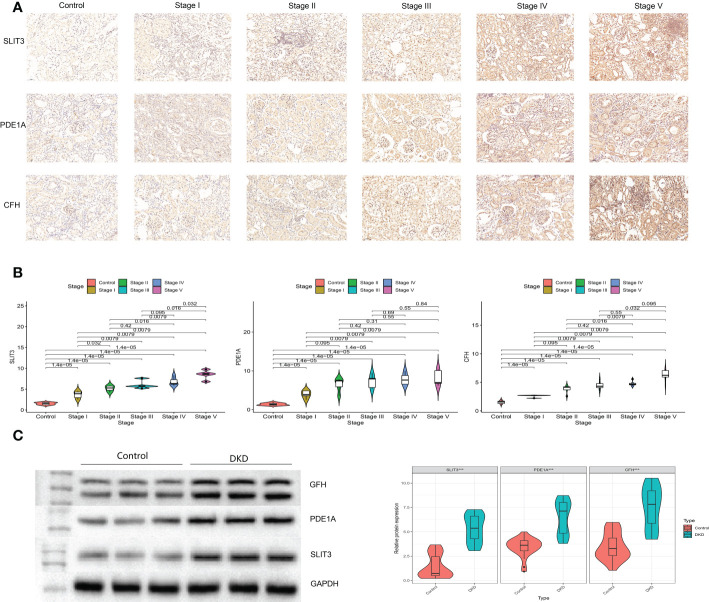
Verification of key genes in clinical participants. **(A)** Immunohistochemistry for control and different stages of DKD participants. **(B)** Quantitative results of immunohistochemistry. **(C)** Western blotting for control and DKD participants.

The plasma levels of CFH, PDE1A, and SLIT3 were evaluated using ELISA. Compared to healthy controls, DKD patients had higher expression of hub genes (**Figure 13A**). Aims to identify the genes associated with DKD development and analyze their association. We found higher CFH, PDE1A, and SLIT3 levels are related to worse DKD development, according to Kaplan-Meier estimations and log-rank tests (CFH: p =0.038; PDE1A: p = 0.025; SLIT3: p = 0.013, **Figure 13B**). Furthermore, the immunohistochemical (IHC) results of kidney tissue showed that compared with the control group, participants with DKD had significant statistical differences in the expression of three key genes, and with the gradual deterioration of DKD, the expression of SLIT3, PDE1A and CFH gradually increased (**Figures 14A, B**). At the same time, we also carried out western blot on the kidney tissue samples, and the results showed the same trend. There were significant statistical differences in the expression of SILT3, PDE1A and CFH between the control group and DKD group (**Figure 14C**). We studied the correlation between ELISA and IHC score using spearman method. The results showed that IHC had a good linear correlation with ELISA (**Figure S3**).

## 4 Discussion

We identified high-variability genes through scRNA-seq strongly influence cell differentiation across homogeneous populations (27). Using cell type identification, scRNA-seq data can be interpreted and cellular heterogeneity can be resolved based on transcriptional and phenotypic interactions (28). By mapping scRNA-seq profiles, we could identify specific types of cells and their marker genes in DKD. There are 8 cell types underwent phenotypic transformation, including Stromal cells, Endothelial cells, NKT, B cells, Epithelial cells, Neutrophils, DC and Macrophages. Among them, macrophages are the most important. Furthermore, we identified 356 different expression genes (DEGs) from the RNA-seq, which are enriched in urogenital system development, kidney development, platelet alpha granule and glycosaminoglycan binding pathways. A weighted gene co-expression network, gene module detection, as well as phenotypic relationships between modules and genes were performed by WGCNA. The highest correlations module is related to regulation of cell adhesion, positive regulation of locomotion, PI3K-Akt, gamma response, epithelial-mesenchymal transition and E2F target signaling pathway. Then we overlapped the DEGs, WGCNA and scRNA-seq, the screened three key genes may be closely related to DKD, which include SLIT3, PDE1A and CFH. Besides, the results of immune infiltration showed that T cells gamma delta, Mast cells resting and Macrophages M2 were positively correlated with the three key genes named SLIT3, PDE1A and CFH, and B cells naive, Neutrophils were negatively correlated with the three key genes. Finally, the three genes SLIT3, PDE1A, and CFH were highly expressed in DKD patients during clinical validation, and low DKD development rates are associated with the high expression of these three genes.

As a result of integrating four datasets, we identified eight cell groups. This was partly similar to the previous research results. A study used GSE131882 dataset for analysis and got the conclusion that in diabetic kidney specimens and controls, 10 cell types were aggregated, including tubular cells, endothelium, parietal epithelial cells, podocytes, collecting duct, mesangial cells, immune cells, distal convoluted tubule, the thick ascending limb, and proximal tubule (20). In our results, macrophages had much stronger intercellular communication than other cells. A significant role is played by macrophages in the development of DKD, causing irreversible changes to the glomeruli and kidney tissue stromal hyperplasia (29). Predominantly M1-containing macrophages characterize diabetes-related kidney injury. In mice lacking the cyclooxygenase-2 gene (Cox-2), M1 was shown to be crucial for the development of DKD. A higher degree of M1 polarization is associated with a higher degree of renal injury in these mice (30). Other publications claimed that the escalating levels of TGF- and galectin-3 in the kidneys of streptozotocin-induced DKD rats indicate a predominate M2 cell type (31). A streptozotocin-induced type 1 diabetic mouse was adoptively transferred with M2 and the reduction of renal damage along with the infiltration of macrophages into the kidney decreased, consisting of interstitial expansion, glomerular hypertrophy and tubular atrophy (32). It has been shown that proxies that promoted M2 polarization, such as Pentraxin-3, can significantly reduce kidney injury in patients with DKD by stimulating the differentiation of M2 macrophages (33). A recent study by Zhang et al. found that inhibiting the activation of M1 macrophages and promoting the transformation of M2 macrophages prevented podocyte damage (34). In a microenvironment mimicking diabetic kidneys, Sirt6 activated M2 and protected podocytes from injury (35). Diabetes pathogenesis is mainly dependent on macrophages, as evidenced by these studies.

Herein, we identified 3 overlap genes of scRNA-seq, DEGs and WGCNA between DKD and control. It has been shown that Slit3 is secreted by M2-like macrophages which are found in adipose tissue and that it stimulates norepinephrine release from sympathetic neurons, which is necessary to adapt to cold environments (36). The osteoprotective role of SLIT3 has been demonstrated in a number of previous studies, indicating that the protein could be underlying targets for therapy in the treatment of metabolic bone disease (37, 38). However, few research illuminated the relationship between Slit3 and DKD. To our knowledge, we are the first team to propose that Slit3 could activate the ECM receptor interaction and focal adhesion pathways. It was deduced that LMWF could reduce ECM expression and inhibite the PI3K/AKT and JAK-STAT pathways, finally improving abnormal human renal mesangial cells (HRMCs) induced by AGEs in DKD (39). Focal adhesion is a crucial factor in maintaining the structural integrity of podocytes (40). Our result indicated that SLIT3 could regulate the ECM receptor interaction and focal adhesion pathways to Influence the pathogenesis of DKD. Strong PDE1A expression was seen in the kidney, suggesting their possible involvement in kidney diseases (41).We found that PDE1A was a key gene of DKD, which is related to focal adhesion and lysine degradation pathways. There are 20 short census repeat domains in the CFH glycoprotein, which binds to chromosome 1q32 and encodes factor H protein, an important inhibitor r of the alternate pathway of complement in the fluid phase of a cell’s surface and in the liquid phase (42). Bonomo et al. (42) indicated that CFH-associated types of mesangial proliferative glomerulonephritis were seen in end-stage kidney disease caused by hypertension or glomerulosclerosis, and fosinopril with CFH demonstrated preferred binding activity. Thus, CFH might be a target for the treatment of mesangial cells associated with DKD (43). Further results of GO and KEGG indicated that the gene is enriched in peroxisome, focal adhesion and lysine degradation pathways. According to Wang, YQ et al., succinate generated by peroxisomes accumulates lipids and ROS in the kidney, causing oxidative stress, and treating DKD and associated metabolic problems by precisely targeting peroxisomal beta-oxidation might be successful. We hypothesized that CFH might cause the peroxisome-generated succinate to be produced, which would lead to DKD and other metabolic diseases. In light of the above evidence, it is clear that there is a close relationship between these three genes and DKD. This hypothesis was also confirmed by our finding that these three genes were not only highly expressed in DKD, but also were closely correlated with renal function development. To confirm the results in a more robust manner, the research sample size must be increased.

In addition, the database used in this work is commonly utilized in other bioinformatics studies. Some scholars have identified VEGFA, NPHs1, WT1, CTGF, SYNPO and POD XL as promising biomarkers to diagnose DKD using GSE30528 and GSE1009 databases (44). Others used GSE30528 database and WGCNA method to identify six key genes related to DKD (22). Using the GSE96804 database, research determined that THBS2, COL1A2, COL6A3, and CD44 might be potential biomarkers and treatment prospects for DKD (45). Finally, about GSE104948, scientists used this database for bioinformatics analysis and experimental verification identified three novel DKD-specific genes (46).

It is found in further studies that T cells gamma delta, neutrophils, B cells naive, mast cells resting, and macrophages M2 may be interrelated cell groups with three key genes in common. It is being investigated whether T-lymphocytes are activated in DKD (47–50). The activation of T-lymphocytes is dependent on two primary signals: the presentation of MHC antigens, and the connection between co-stimulatory molecules B7-1 and CD28, resulting in T lymphocyte activation or repression. There are a variety of ways in which T-lymphocyte activation can occur in DN, including systemic reactions or local reactions due to MHC class II influenced by podocytes (51), mediated by B7-1, which is responsible for the injury of podocytes in DKD (47, 52). It is important in the early stages of DN to activate TNF-α signaling pathways and to activate T-lymphocyte immunity. A moderate albuminuria is initiated by both pathways, and it is likely that both pathways affect the development of moderate albuminuria as well as the impairment of renal function. The T-lymphocyte immune markers sCTLA-4 and CD28 involved in the pathogenesis and advancement of DN (50). M2 macrophages play a controversial role in kidney tissue fibrosis, since they can partake in the restoration and remodeling, differentiate into fibroblasts, as well as stimulate myofibroblast proliferation and promote of DN kidney injury through phagocytosis, restraining the toxic effects of T cells, downregulation of inflammatory cytokines and chemokines, and recovery from shock (53). Researchers have discovered that M1 macrophages have an early stage of renal damage and an M2 phenotype when the kidney is in the repair stage. Furthermore, M1 macrophages can eventually become M2 macrophages(2011). DN could be treated by stimulating M2 macrophages and inhibiting M1 macrophage numbers. Neutrophils are the main cause of acute kidney injury, according to current research (54), and their role in DKD was revealed by Wang, YJ (55). The result conducted by Wang, YJ showed that resting mast cells, macrophages M1/M2, infiltrated immune cells and T cells CD8, were significantly related to DKD (55), which was consistent with ours. Li, T et al. proposed that regulation B cells are significantly reduced in DKD sample compared with diabetic controls and healthy, thus supporting the conjecture that regulatory B cells has an impact on the disease (56). This conclusion agree with those of a study that performed reduced numbers of regulatory B cells in patients with primary glomerulonephritis (57). Herein, we are the first team to propose the hypothesis that naive B cells are negatively related to three key genes.

To explain the involvement of the core genes in DKD patients to further clarify the regulatory relations. We found the PTF1A was annotated to the most significantly enriched motifs for the 3 key genes. There are several helix-loop-helix (bHLH) proteins that are specifically expressed in the brain, spinal cord, retina, pancreas, and enteric nervous system, including transcription factor Ptf1a. Assembling Ptf1a with an E protein and RBPJ (or RBPJl) results in a transcription trimeric complex PTF1. The function of Ptf1a in pancreatic development is to regulate multipotent progenitor cell proliferation and acinar cell maintenance (58). Exome sequences reveal a mutation in PTF1A in a family with multiform cases of early-onset diabetes (59, 60). According to Olcay et al., a homozygous PTF1A enhancer mutation caused an isolated pancreas agenesis in two neonatal diabetes patients (61). In early neonatal periods, patients with PTF1A distal enhancer mutations present with IUGR, indicating *in utero* insulin deficiency (62; 61, 63–66). It indicated that different mutations in enhancers may show genotype-phenotype correlations (63). It indicated that PTF1A has an impact on DKD. In spite of this, it remains unclear how identical mutations at the distal enhancer affect PTF1A function.

Additionally, since there is no experimental verification of the DKD key genes, the results are probably inaccurate, Therefore, bioinformatics analysis needs to be combined with GeneCards database information regarding human DKD. It is displayed that the level of key genes is significantly correlated to the expression level of several DKD related genes. Especially, SLIT3 and PDE1A were strongly associated with INSR and HNF1B, respectively. Insulin receptor encoded by INSR interacts between extracellular and intracellular signaling pathways, and is necessary to insulin action. Diabetes type 2 may be associated with INSR, which involved in adipogenesis and beta-cell insulin secretion (67, 68). Further, INSR expression changes in the kidney during diabetes (69), indicating that it contributes to DKD of type 2. Diabetes secondary to mutations in HNF1B was first showed in a Japanese family in 1997 (70), increasing documentation of the phenotype associated with this mutation has been published since then. There has been evidence that changes in the HNF1B gene contribute to a slight predisposition to type 2 diabetes, Possibly caused by mutations in the HNFB1 gene that create a protein that is incapable of binding DNA or fails to transactivate DNA following binding (71). It is clear from these evidences that key genes are important in the pathogenesis of DKD, as well as potential diagnostic and therapeutic targets (44, 72, 73).

Our research has some limitations. First, there were too few PBMC data sets of DKD. At present, only GSE185011 and GSE142153 were found, among which GSE185011 samples were too few. Therefore, we can’t verify the expression of three key genes in other data sets. We look forward to the publication of a larger data set to further verify the expression of the three key genes we have obtained. A second limitation is that the relationship between the genes and cell types studied thus far has not been verified through other functional studies or *in vitro* studies, which will be the focus of our future research. The third shortcoming is that in order to get more differential genes, we set the threshold to 0.585, which seemed to be a bit low, but can get more differential genes, and might exaggerate the role of some genes in the disease. Finally, Due to ethical problems, we couldn’t obtain completely normal renal tissue samples. In order to further set up the control group, we could only choose subjects with relatively healthy kidney participants. These subjects might have slight abnormalities, but there was no overlap with the DKD studied in terms of mechanism. Because the mechanisms did not coincide and there was no clinical correlation, we thought the results were still reliable. Of course, this is also a project that we will carry out in the future. We will further obtain tissue samples of healthy kidneys from paracancerous tissue of patients with renal cancer for further.

## 5 Conclusion

As a result of our innovative single-cell and transcriptome analyses, we discovered relations between three genes and DKD, as well as identified transcriptional regulators and intercellular pathways involved. Our next step was to clarify the effect of macrophages on DKD. Three new targets have been identified for future translational studies on DKD based on our novel mechanism of DKD.

## Data availability statement

Publicly available datasets were analyzed in this study. This data can be found here: GSE131882, GSE1009, GSE30528, GSE96804 and GSE104948. https://www.ncbi.nlm.nih.gov/geo/info/datasets.html.

## Ethics statement

This study was approved by the Ethics Committee of People’s Hospital of Xinjiang Uygur Autonomous Region (Num. KY2020101945). The patients/participants provided their written informed consent to participate in this study.

## Author contributions

XZ, HJ, and CL were responsible for putting forward scientific problems and preparing all experiments. PC is responsible for all data analysis and data visualization. LZ and LX mainly undertook ELISA experiments. XC was mainly responsible for the collection and follow-up of patients. SW and MW was mainly responsible for writing manuscripts. All authors contributed to the article and approved the submitted version.
