# Supplementary material for: Single-cell RNA and transcriptome sequencing profiles identify immune-associated key genes in the development of diabetic kidney disease
Source: Front Immunol. 2023 Mar 29;14:1030198. doi: 10.3389/fimmu.2023.1030198 (PMC10091903; doi:10.3389/fimmu.2023.1030198)
Supplement: Supplementary file 1 [file DataSheet_1.docx]

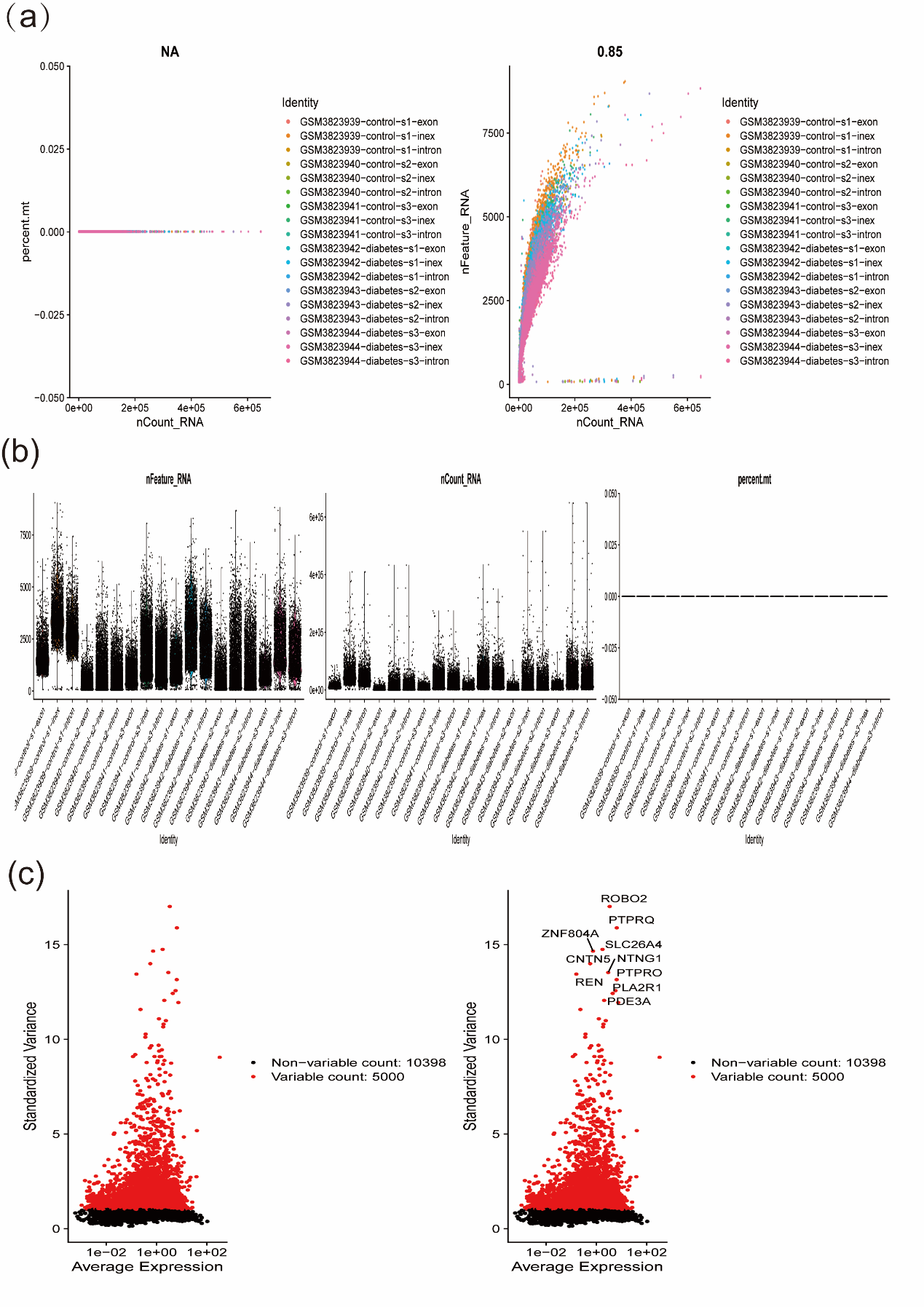


Figure S1. Expression profile data of single cell. (a) The relationship between RNA count and mitochondrial RNA ratio and gene number. (b) Violin plots performing the level of genes. (c) Screening the 5000 highly variable genesacrosscells according to single cell RNA-seq data. Black dots show non-variable genes and red dots show the highly variable genes.


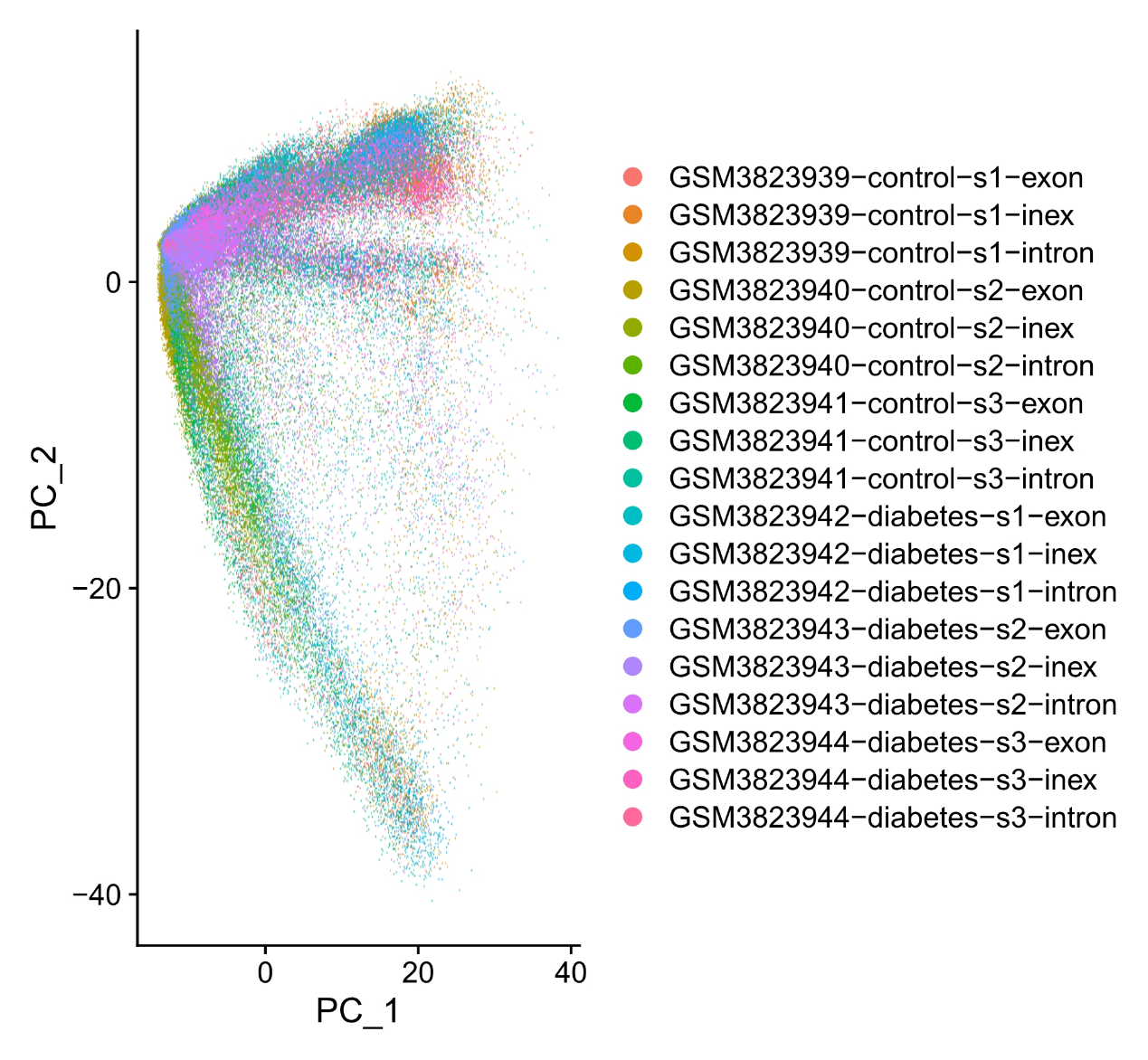


Figure S2. Principal component analysis of 20 genes in the samples.


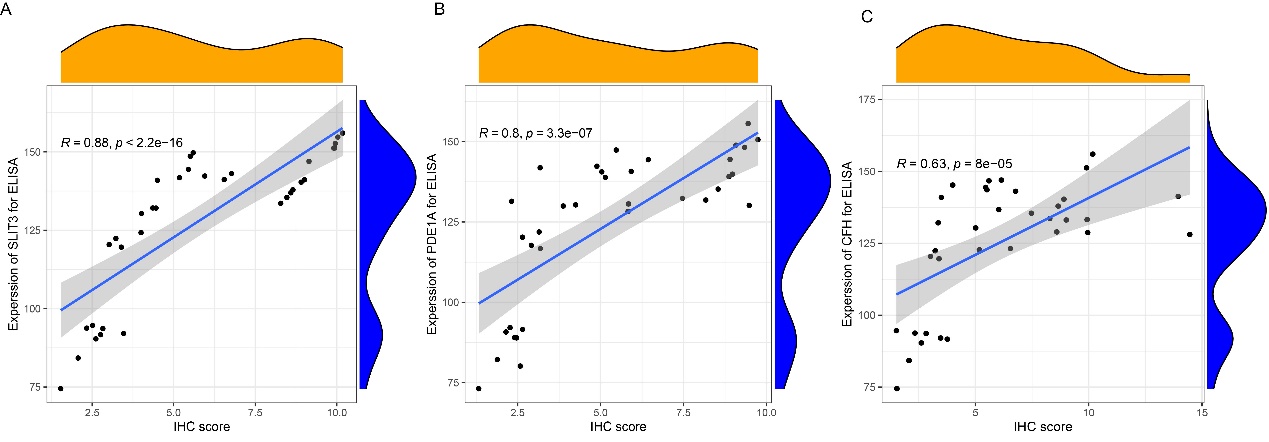


Figure S3.Correlation between expression of SLIT3, PDE1A, CFH for ELISA and IHC scores.


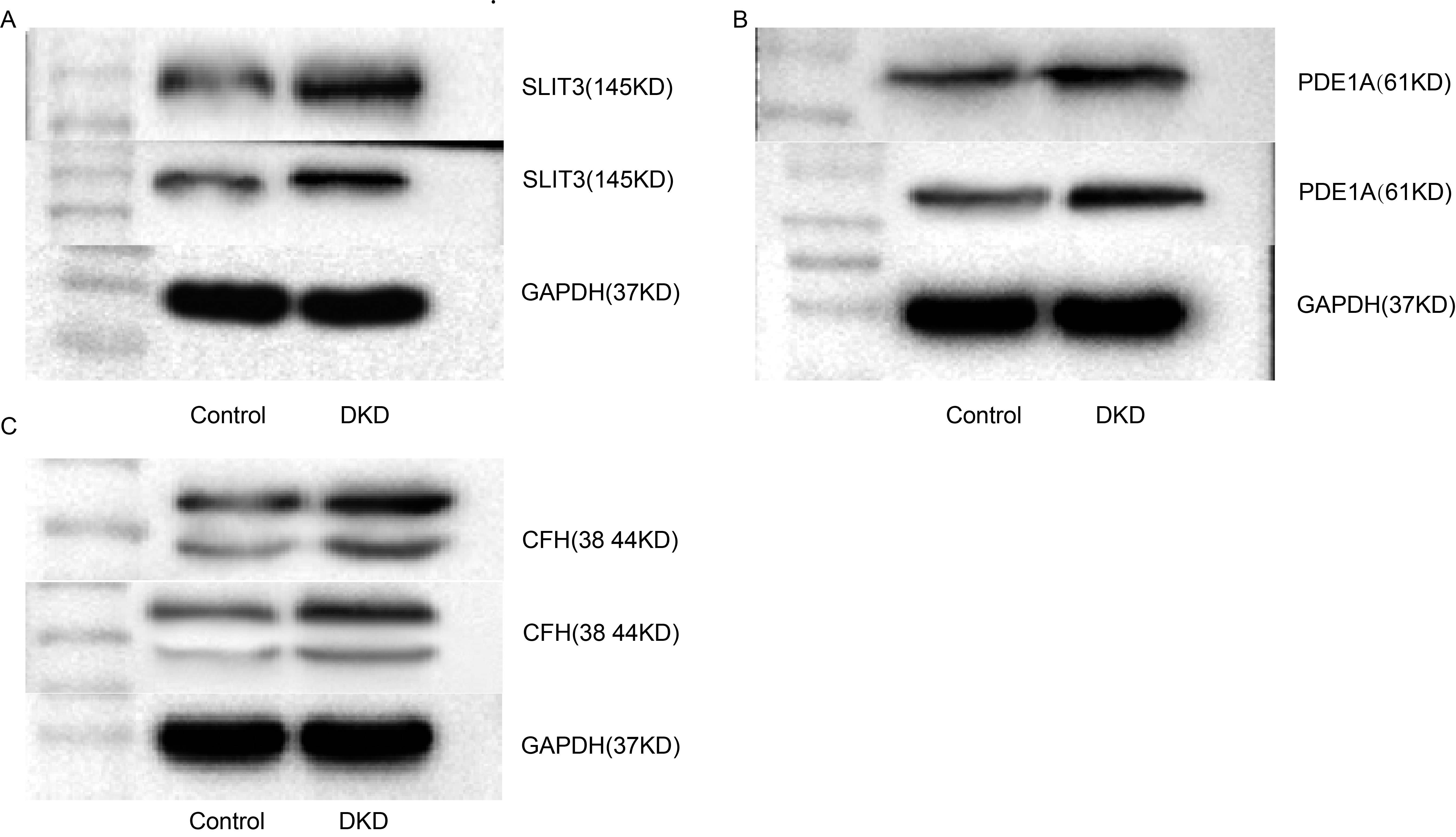


Figure S4. Western blotting for control and DKD participants(The verifications of protein levels in kidney tissues was repeated at least three per group).
